# Effects of ginger intervention on anthropometric and body composition indices in patients with type 2 diabetes: a GRADE-assessed systematic review and meta-analysis of randomized controlled trials

**DOI:** 10.1186/s12906-026-05492-2

**Published:** 2026-08-05

**Authors:** Ali Keshavarz, Aylee Sobhanifar, Parna Alizadeh, S Faezeh Hashemi Moghaddam, Monirsadat Mirhoseini, Fatemeh Borazjani

**Affiliations:** 1 Student Research Committee, Ahvaz Jundishapur University of Medical Sciences, Ahvaz, Iran; 2 Nutrition and Metabolic Diseases Research Center, Clinical Sciences Research Institute, Ahvaz Jundishapur University of Medical Sciences, Ahvaz, Iran; 3 Student Research Committee, Tabriz University of Medical Sciences, Tabriz, Iran; 4 Student Research and technology Committee, Faculty of Health and Nutrition, , Bushehr University of Medical Sciences, Bushehr, Iran; 5 Department of Clinical Nutrition, School of Nutrition and Food Sciences, Shiraz University of Medical Sciences, Shiraz, Iran; 6 Department of Cellular and Molecular Nutrition, Faculty of Nutrition and Food Technology, Shahid Beheshti University of Medical Sciences, Tehran, Iran; 7 Department of Nutrition, School of Allied Medical Sciences, Ahvaz Jundishapur University of Medical Sciences, Ahvaz-Iran-Golestan Highway, Ahvaz, 6135715794 Iran

**Keywords:** Ginger, Type 2 diabetes, Anthropometry, Body composition, Meta-analysis

## Abstract

**Background and aims:**

Evidence on the effects of ginger supplementation on anthropometric and body composition indices in adults with type 2 diabetes mellitus (T2DM) is inconsistent, and the certainty of this evidence has not been fully evaluated. This study aimed to systematically review and meta-analyze randomized controlled trials assessing these effects.

**Methods:**

PubMed, Scopus, and Web of Science were searched until October 5, 2025. We used random-effects meta-analysis to find average differences with 95% CIs. Study quality and evidence certainty were checked with Cochrane RoB-2 tool and GRADE approach. Subgroup, meta-regression, sensitivity, and publication bias analyses were also done.

**Results:**

This meta-analysis included 692 participants from 12 RCTs. Ginger supplementation significantly reduced body mass index **(**BMI) (− 0.24 kg/m²; 95% CI: −0.48, − 0.00; *p* = 0.043) and body fat percentage (BF%) (− 1.44%; 95% CI: −2.49, − 0.38; *p* = 0.007), while no significant effects were observed on body weight (BW), waist circumference (WC), hip circumference (HC), or waist-to-hip ratio (WHR). Bias assessment showed 9 studies with some concerns, 1 low, and 2 high risk. Evidence quality was moderate for 4 outcomes (BMI, WHR, HC, BF%), low for WC, and high for BW.

**Conclusion:**

Ginger reduced BMI and BF%, with no significant change in BW, WHR, HC, or WC.

**Trial registration:**

This study was prospectively registered in PROSPERO (CRD420251162956).

**Supplementary Information:**

The online version contains supplementary material available at https://doi.org/10.1186/s12906-026-05492-2.

## Introduction

Type 2 diabetes (T2DM) is a major metabolic disorder [1]. The global diabetic prevalence for adults aged 20–79 years was 10.5% (536.6 million people) in 2021, according to the report, and is projected to reach 12.2% (783.2 million) by ‍‌‍‍‌2045 [2]. This disease necessitates designing and implementing strategies for its prevention, management, and treatment, which makes it one of the main health concerns worldwide [3].

T2DM remains a complex metabolic disorder, mainly involving high blood sugar, impaired β-cell function, and insulin resistance in body cells [4, 5]. The etiological factors contributing to its development have long been debated [6]. In recent years, studies have shown that the major causes of the rising disease prevalence are increasing obesity rates, sedentary lifestyle, and unhealthy dietary patterns [6–8]. These factors may escalate insulin resistance. Consequently, individuals may be exposed to a wide range of complications, including inflammation, fatty liver, cardiovascular dysfunction, dyslipidemia, retinopathy, neuropathy, and nephropathy [9–11]. Besides, excess adiposity is a major determinant that elevates the lifetime risk of T2DM for males from 7% to 70% and for females from 12% to 74% with the increase of BMI [12]. One of the main methods to solve the problem of T2DM is the effective management of obesity and body composition [13]. At present, modifications in lifestyle, medicines, and bariatric surgeries are the options that have been indicated to reduce diabetes incidence and enhance glycemic control [14, 15]. A weight loss of 5% to 10% may result in substantial metabolic changes and even the remission of diabetes in some cases [16].

Ginger‍‌‍‍‌ *(Zingiber officinale)* is a medicinal herb that has various natural compounds which include phenolic compounds, terpenes, polysaccharides, organic acids, and fibers. As a complementary therapy, it may improve anthropometric indices in patients with T2DM [17, 18].

Several trials explored the impact of ginger on body weight (BW), body mass index (BMI), body fat percentage (BF%), and waist circumference (WC) in individuals with T2DM; however, the results remain inconsistent [18–23]. At present, there is no standardized guideline concerning the dosage or the duration of ginger supplementation for obtaining the beneficial effects or for impeding the possible limitations in individuals with T2DM [24, 25]. Given the growing global interest in ginger as a potential adjunct therapy in diabetes [26], summarizing all available evidence through a rigorous quantitative synthesis is necessary. Hence, this systematic review and meta-analysis was conducted to comprehensively assess the impact of ginger supplementation on anthropometric and body composition indices in patients with T2DM.

## Methods

### Study protocol and registration

This systematic review and meta-analysis was conducted in accordance with the PRISMA 2020 guidelines [27], and had its protocol registered in PROSPERO (CRD420251162956) with Ethics Committee approval (IR.AJUMS.REC.1404.569). Based on the PICOS framework [28], the study focused on adult patients with type 2 diabetes (T2DM) (Population) who were given ginger supplements (Intervention). They were compared to placebo or control groups (Comparison). BW, BMI, WC, WHR, HC, and BF% were the assessed outcomes (Outcomes). The literature search was limited to clinical trials only (Study ‍‌‍‍‌Design).

### Search strategy

An extensive systematic search of PubMed, Web of Science, and Scopus was performed for records from the inception of the databases to October 5, 2025. A combination of Medical Subject Headings (MeSH) and non-MeSH phrases describing ginger (*Zingiber officinale*) and diabetes was utilized along with Boolean operators (AND/OR) in the title and abstract fields. No restriction was imposed on the publication year. Reference lists of the identified articles and reviews were checked manually to find other potential studies. Comprehensive search strategies and outcomes of each database are presented in Supplementary Table S1.

### Eligibility criteria

All records were independently screened by two reviewers (P.A. and A.K.) at the title/abstract and full-text levels, with disagreements resolved by consensus or consultation with a third reviewer (F.B.). Eligible studies were randomized controlled trials in adults with type 2 diabetes mellitus that evaluated oral ginger (Zingiber officinale) supplementation compared with placebo, no intervention, or standard care, and reported at least one anthropometric or body composition outcome (e.g., BW, BMI, WC, HC, WHR, or BF%). Non-randomized studies, uncontrolled trials, multifactorial interventions, studies involving children or adolescents, and those lacking sufficient quantitative data were excluded, as were animal, in vitro, review, and conference publications.

### Data extraction and management

Data extraction was carried out independently by two reviewers (A.S. and S.F.H.M.) who used a standardized form. They resolved disagreements through discussion or, if necessary, a third reviewer (F.B.) was involved.

Data extracted included characteristics of studies and participants, details of interventions, and anthropometric and body composition outcomes as reported (BW, BMI, WC, HC, WHR, BF%). When available means and SDs at baseline and follow-up, or changes from baseline, were documented.

Standardizing, enabling inter-study comparisons, and allowing further meta-analytical calculations were the reasons for putting all extracted data in elaborately arranged tables.

### Risk of bias and certainty of evidence

Two reviewers (A.K. and P.A.) independently assessed the risk of bias of the included randomized trials using the Cochrane Risk of Bias version 2 (RoB-2) tool [29]. When there was a difference of opinion, the two reviewers discussed the issues and if the situation required the third reviewer (F.B.) was consulted. The assessment was based on the five domains of RoB-2: randomization process, deviations from intended interventions, missing outcome data, measurement of the outcome, and selection of the reported result.

The overall certainty of evidence for each outcome result was determined by the GRADE approach [30]. The certainty level was evaluated from a combination of five domains: risk of bias (which was informed by RoB-2), inconsistency, indirectness, imprecision, and publication bias. The final judgment was in one of four categories: high, moderate, low, or very low. The findings were compiled in a GRADE evidence profile table (Table 2).

### Statistical analysis

Comprehensive Meta Analysis (CMA) software, version 3 (Biostat, Englewood, NJ, USA) [31], was used to perform all statistical analyses. For each outcome, the effect size was calculated as the weighted mean difference (WMD) with corresponding 95% confidence intervals (CIs). Mean changes were obtained by subtracting baseline from post intervention values for both the intervention and control groups.

If studies didn’t report change scores or SDs, the SDs of the mean change were calculated using this formula [32]:

$$\begin{array}{ll}\mathrm{SD}=\text{square root}\\\left[\left(\text{SD baseline}\right)^2+\left(\text{SD final}\right)^2-\left(2\mathrm{R}\times\text{SD baseline}\times\text{SD final}\right)\right]\end{array}$$

Assuming a correlation coefficient (R) of 0.5 [33]. In studies reporting standard errors (SEs) instead of SDs, SDs were calculated as SE × square root (n), where n is the number of participants in each group. When outcome data were reported as medians with ranges, mean values and SDs were estimated using the method described by Hozo et al. [34].

Between-study heterogeneity was assessed using Cochran’s Q test and quantified with the I² statistic. An I² value ≥ 50% or a *p*-value < 0.05 for Cochran’s Q was considered indicative of substantial heterogeneity. A random-effects model (DerSimonian and Laird method) was used from the start to account for differences between studies, regardless of the I² values [35, 36].

We performed predefined subgroup analyses to investigate potential sources of heterogeneity based on baseline BMI (≤ 29.9 vs. >29.9 kg/m²), intervention duration (≤ 8 vs. >8 weeks), and ginger dose (< 2 vs. ≥2 g/day). These subgroup thresholds were defined a priori. The BMI cut-off was selected to distinguish participants with overweight from those with obesity according to standard clinical classifications. The intervention duration threshold of 8 weeks was chosen because it represented the most common treatment duration among the included trials and provided a balanced distribution of studies between subgroups. The ginger dose threshold of 2 g/day was selected based on the doses most frequently evaluated in previous randomized trials and meta-analyses, allowing clinically meaningful comparisons [37, 38]. Between-subgroup differences were assessed using the Q statistic for heterogeneity.

To further examine dose–response and time–response relationships, random-effects meta-regression analyses were performed using the unrestricted maximum likelihood method, with ginger dose (g/day) and intervention duration (weeks) entered as continuous moderators.

We checked how stable the pooled results were using a leave-one-out sensitivity analysis, removing each study one at a time to see its effect [39].

Publication bias was checked with funnel plots and tested using Begg’s, Egger’s, and fail-safe N methods [40]. In all analyses, *p*-values < 0.05 were considered statistically significant.

## Results

### Search results and study selection

**Fig. 1:**
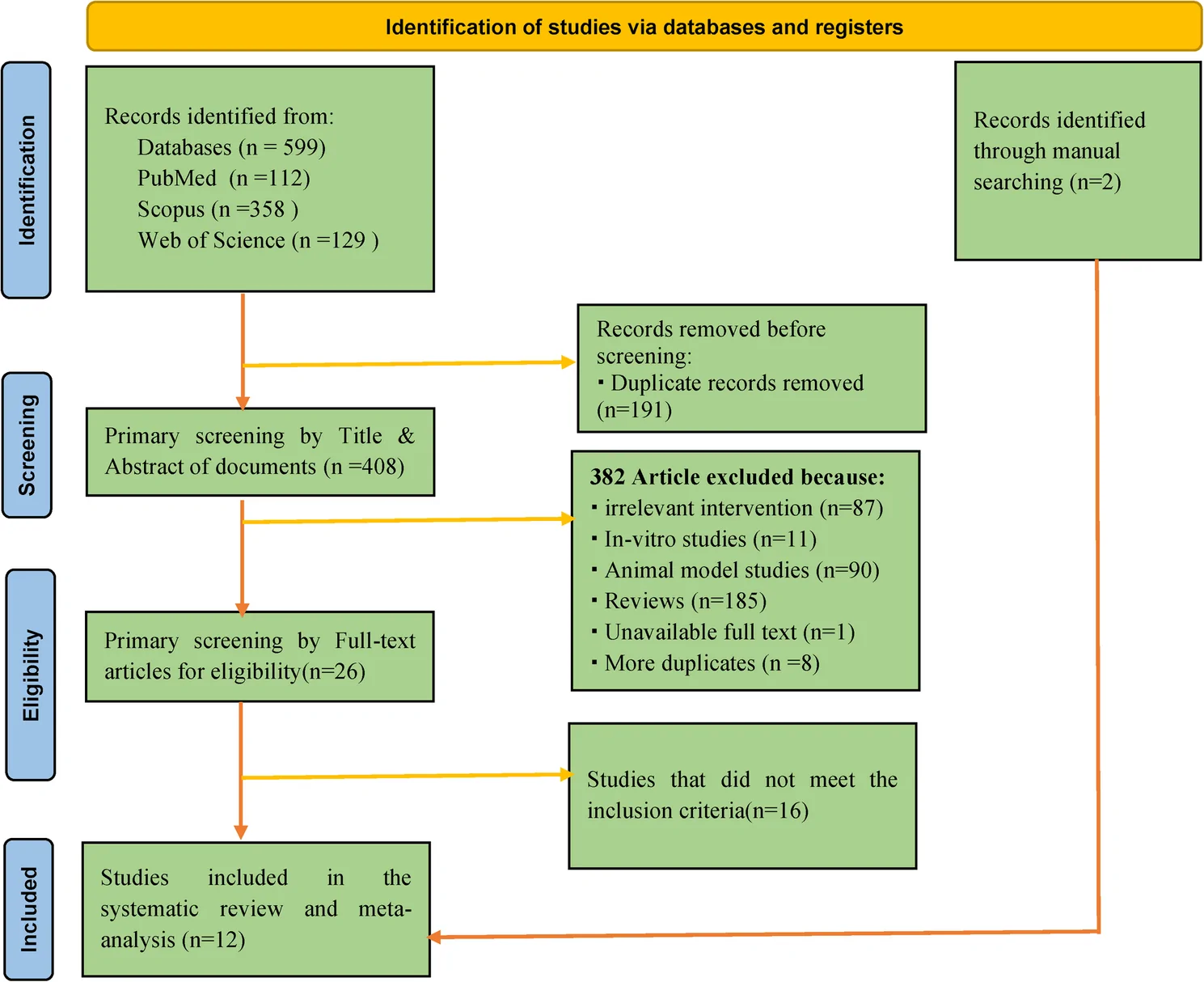
PRISMA flow chart of the study selection process for inclusion studies in the systematic review and meta-analysis

A total of 601 records were identified, 599 from electronic databases (PubMed, *n* = 112; Scopus, *n* = 358; Web of Science, *n* = 129) and 2 from manual searches of reviews and reference lists. Of the 599 database records, 191 duplicates were removed. After removing 191 duplicates, 408 articles were brought to the first screening stage based on titles and abstracts. At this point, 382 records were rejected for the following reasons: irrelevant intervention (*n* = 87), in vitro design (*n* = 11), animal studies (*n* = 90), review articles (*n* = 185), unavailable full text (*n* = 1), and duplicates (*n* = 8). The full texts of 26 articles were assessed for eligibility. Sixteen of the publications were excluded during this stage mainly because of their failure to meet the inclusion criteria (e.g., non-trial study, no control group, combined interventions, or insufficient outcome data). Moreover, the duplicate publications of the same study with the same datasets were identified, and only the more detailed publication was retained in order to avoid duplication bias. Consequently, this systematic review and meta-analysis included 12 randomized controlled trials (RCTs). Fig 1 presents the PRISMA flow diagram of the study selection process.

### Characteristics of included studies

Table 1 summarizes the characteristics of the 12 RCTs (14 eligible intervention arms) included in the meta-analysis, including details on study location, participant characteristics, sample size, intervention type and dosage, duration, and measured outcomes. The trials were published between 2013 and 2023, mostly from Iran [22, 37, 41–48], with one trial from Brazil [21], and one from Egypt [49]. The powdered ginger (capsule), tablet, extract, or combination with exercise was the form of ginger supplementation, at a dosage of 1–3 g/day for 4–12 weeks. BW, BMI, WC, HC, WHR, and BF% were assessed. To maintain blinding, placebos containing inert materials (e.g., wheat flour, starch, microcrystalline cellulose, black tea, or lactose) were used.

**Table 1:** Characteristics of the studies included in the systematic review and meta-analysis

Author, year (Ref)	Country	Patient status	Sample Size (I/ C)	BMI (kg/m2)	Mean age, (year)	Women (%)	Study design	Follow up duration (weeks)	Intervention )Ginger type(	Placebo	Ginger Dose (g/d)	Outcome(s)
Arablou, 2014 [41]	Iran	T2DM	I:33 C:30	I: 26.9 ± 3.6 C:26.8 ± 3.4	I: 52.6 ± 8.4 C:52.0 ± 9.0	I:75.8 C:76.7	DB, PC RCT	12	Powder Caps (Rhizome)	Wheat Flour Caps	1.6	BW, BMI
Honarvar, 2019^*^ [22]	Iran	T2DM	I:23 C: 22	I: 29.9 ± 3.5 C: 29.2 ± 4.6	I: 51.7 ± 8.5 C:49.6 ± 8.6	I: 65.2 C:72.7	DB, PC RCT	10	Grinded Powder Caps	Wheat Flour Caps	2	BW, BMI, HC, WC, WHR
Askari, 2019 a [42]	Iran	Obese F with T2DM	I: 15 C:15	I: 34.1 ± 3.1 C: 30.5 ± 2.0	I:50.4 ± 2.7 C:48.3 ± 8.2	100	RCT	12	Powder Caps (Rhizome)+HICT	Wheat Flour Caps +HICT	1.6	BMI, BW, WHR BF%
Askari, 2019 b [42]	Iran	Obese F with T2DM	I:15 C:15	I:31.0 ± 3.2 C:31.7 ± 3.7	I:49.1 ± 3.3 C:50.1 ± 3.3	100	RCT	12	Powder Caps (Rhizome)+MICT	Wheat Flour Caps +MICT	1.6	BMI, BW, WHR BF%
Azimi, 2016 [43]	Iran	T2DM	I:41 C: 39	I: 29.05 ± 0.2 C:28.40 ± 0.2	I: 55.2 ± 1.1 C:53.6 ± 1.3	I:63.4 C: 61.5	SB, PC RCT	8	Powder + Black Tea	Black tea	3	BW, BMI, WC
Carvalho, 2021 [21]	Brazil	T2DM	I:9 C:12	I: 29.5 C:27	57.9 ± 9.2	66.7	DB, PC RCT	4	Dry Extract Caps (0.1%)	Microcell. Cellulose Caps	1.2	BW, BMI, WC, HC, WHR
Gayar, 2019 [49]	Egypt	Newly Dx Obese withT2DM	I:40 C:40	I: 32.3 ± 1.5 C: 32.3 ± 1.4	I: 46.3 ± 9.5 C:46.1 ± 8.7	I:52.5 C: 45.0	SB, PC RCT	8	Powder (Dried Rhizome)	Wheat Flour Caps	1.8	BMI
Ghoreishi, 2023 [44]	Iran	T2DM With NAFLD	I:36 C:36	I: 30.9 ± 4.2 C: 31.3 ± 4.9	I:51.2 ± 9.0 C:56.8 ± 7.3	I:66.7 C:75	DB, PC RCT	12	Powder Caps	Starch/Ginger (10:1) Caps	2	BW, BMI, WC, HC, WHR
Mahluji, 2013 [45]	Iran	T2DM	I:28 C:30	I:29.1 ± 3.1 C:29.6 ± 4.8	I:49.2 ± 5.1 C:53.1 ± 7.9	I:46.2 C:42.0	DB, PC RCT	8	Powder Tab (Rhizome)	Corn starch Tab	2	BW, BMI
Mozaffari-Khosravi, 2014 [46]	Iran	T2DM	I:40 C:41	I:28.1 ± 5.3 C:28.5 ± 5.0	I: 49.8 ± 7.2 C:51.0 ± 7.7	I:67.5 C:56.1	DB, PC RCT	8	Powder Caps	Microcell. Cellulose Caps	3	BMI
Mardanpour-Shahrekordi, 2017 a [47]	Iran	Mid-age Obese W with T2DM	I:12 C:10	I: 32.6 ± 1.8 C:34.6 ± 4.9	45–60	100	RCT	10	Extract Caps	Roasted Wheat Flour Caps	1	BW, BMI, BF%, WC, HC, WHR
Mardanpour-Shahrekordi, 2017 b [47]	Iran	Mid-age Obese W with T2DM	I:12 C:12	I: 34.0 ± 4.3 C:31.5 ± 2.0	45–60	100	RCT	10	Extract Caps+ AT	AT	1	BW, BMI, BF%, WC, HC, WHR
Shidfar, 2015 [48]	Iran	T2DM	I:22 C:23	I:29.5 ± 2.8 C:29.2 ± 3.1	I: 45.2 ± 7.6 C:47.1 ± 8.3	NR	DB, PC RCT	12	Powder Caps	Lactose Caps	3	BW, BMI, WC
Veisi, 2023 [37]	Iran	T2DM	I:20 C:21	I: 26.5 ± 3.7 C: 28.4 ± 4.4	I:60.1 ± 11.1 C:59.6 ± 10.7	I:50 C:45.5	DB, PC RCT	8	Powder Caps	Starch Caps	2	BW, BMI, WC, HC, WHR

*AT* Aerobic Exercise Training, *BF% *Body Fat Percentage, *BW* Body Weight, *BMI* Body Mass Index, *C* Control, *Caps* Capsules, *DB* Double-Blinded, Extract Caps: Ginger Extract Capsules, *HICT* High-Intensity Concurrent Training, *HC* Hip Circumference, Microcell. Cellulose: Microcrystalline Cellulose, *MICT* Moderate-Intensity Concurrent Training, *I* Intervention, Mid-Age: Middle-Aged, Newly Dx Obese: Newly Diagnosed Obese Patients, *NIDDM* Non-Insulin Dependent Diabetes Mellitus, *NR* Not Reported, *Obese F* Obese Females, *Obese W* Obese Women, *PC RCT* Placebo-Controlled Randomized Clinical Trial, *Powder Caps* Powdered Ginger Capsules, *RCT* Randomized Controlled Trial, *SB* Single-Blinded, Supplement: Ginger Supplementation, *Tab* Tablet, *T2DM* Type 2 Diabetes Mellitus, *WC* Waist Circumference, *WHR* Waist-To-Hip Ratio, *With Ex* Alongside Exercise

*SE values reported by Honarvar et al. [22] were considered as SD due to statistical implausibility and inconsistency with Zarezadeh et al. [50]

### Risk of bias and quality assessment

**Fig. 2:**
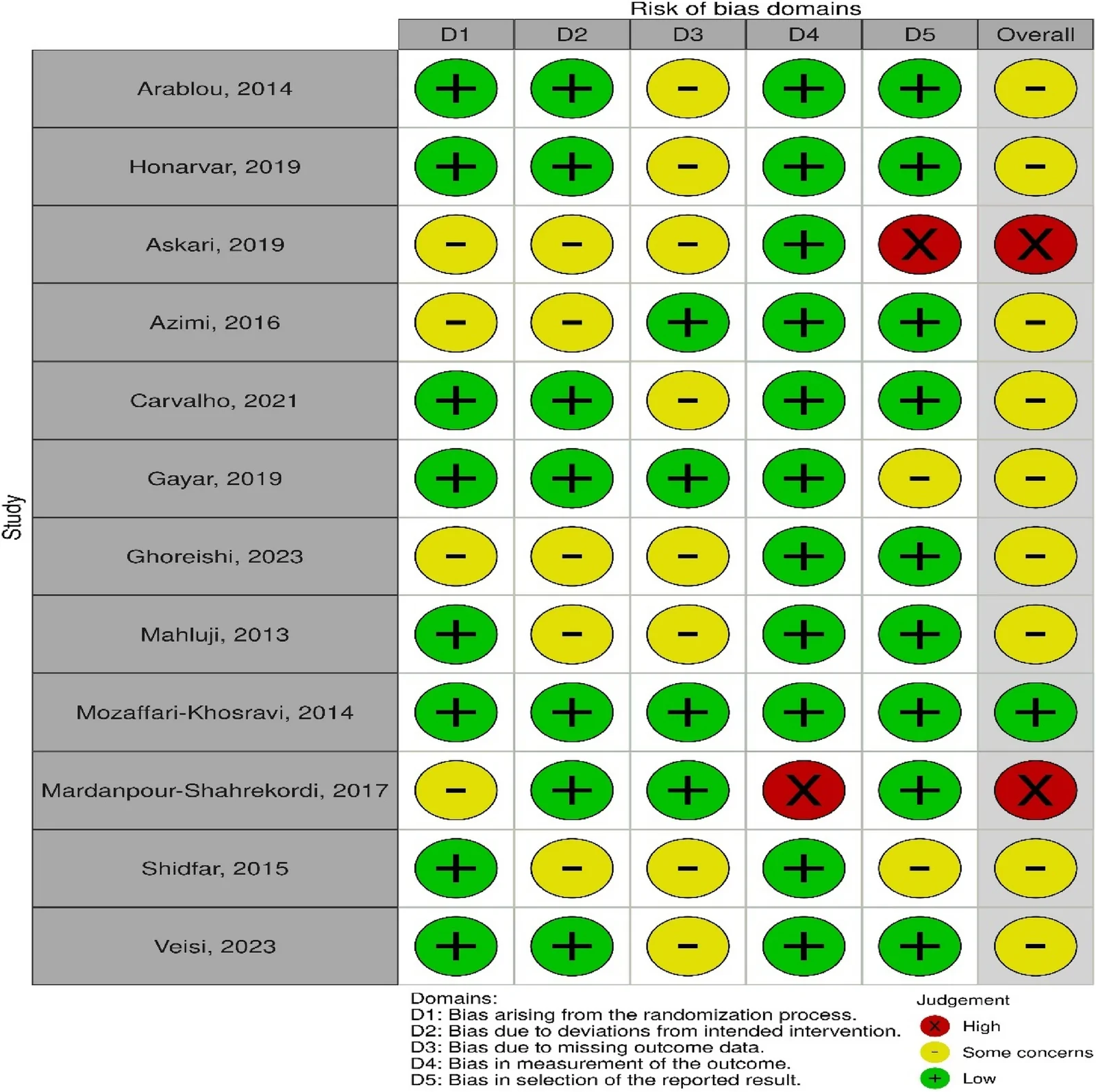
Quality assessment of studies according to Cochrane risk-of-bias 2 (RoB-2)

According to RoB-2, 9 studies showed some concerns [21, 22, 37, 41, 43–45, 48, 49], 2 were high risk [42, 47], and 1 was low risk of bias [46]. Fig. 2 presents the detailed risk-of-bias assessment across the five domains of the Cochrane RoB-2 tool for each included study. In D1 (randomization process), most studies performed well, with 8 trials rated as “Low risk” and 4 trials showing “Some concerns”. Similarly, in D2 (deviations from intended interventions), 7 studies demonstrated “Low risk”, while 5 studies had “Some concerns”. The most frequent issues were observed in missing outcome data (D3), where 8 trials showed “Some concerns”, and in measurement of the outcome (D4), with 1 study rated as having “High risk”. Moreover, selective reporting (D5) was identified as a notable source of bias, with one study judged as “High risk” and two studies categorized as having “Some concern”.

Beyond the study-level risk of bias evaluation, the certainty of evidence was assessed at the outcome level using the GRADE framework. As summarized in Table 2, the certainty of evidence ranged from high to low across outcomes, with downgrading mainly driven by inconsistency, imprecision, and, for selected outcomes, risk of bias or publication bias.

**Table 2:** GRADE summary of evidence for ginger supplementation and anthropometric outcomes in type 2 diabetes

Certainty assessment						Summary of findings		Quality of evidence
Outcomes	Risk of bias	Inconsistency	Indirectness	Imprecision	Pub bias	No. of patients Intervention/Control	WMD; 95%CI	
Body Weight (BW)	No serious limitations	No serious limitations	No serious limitations	No serious limitations	No serious limitations	266/265	−0.02 kg; −0.15, 0.10	⊕⊕⊕⊕ High
Body Mass Index (BMI)	No serious limitations	Serious limitations	No serious limitations	No serious limitations	No serious limitations	346/346	−0.24 kg/m²; −0.48, − 0.00	⊕⊕⊕🞅 Moderate
waist-to-hip ratio (WHR)	No serious limitations	No serious limitations	No serious limitations	Serious limitations	No serious limitations	142/143	0.003; −0.006, 0.011	⊕⊕⊕🞅 Moderate
Waist Circumference (WC)	No serious limitations	No serious limitations	No serious limitations	Serious limitations	Serious limitations	175/175	−0.06 cm; −0.61, 0.49	⊕⊕🞅🞅 Low
Hip Circumference (HC)	No serious limitations	No serious limitations	No serious limitations	Serious limitations	No serious limitations	112/113	0.00 cm; −0.33, 0.35	⊕⊕⊕🞅 Moderate
Body Fat Percentage (BF%)	Serious limitations	No serious limitations	No serious limitations	No serious limitations	No serious limitations	54/52	−1.44%; −2.49, − 0.38	⊕⊕⊕🞅 Moderate

### Main outcomes

#### Effects of ginger supplementation on BW

**Fig. 3:**
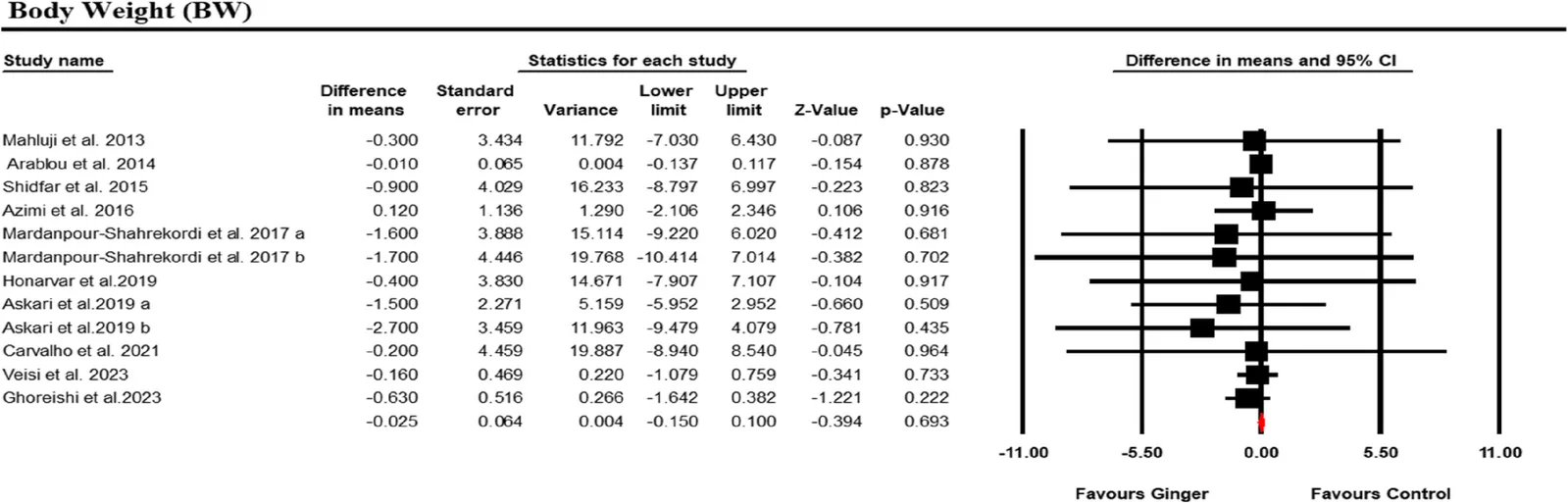
Forest plot of weighted mean difference (95% CI) for ginger effect on body weight

Figure 3 presents the forest plot for BW, showing the WMD and 95% CIs for each study and the overall pooled estimate. The pooled effect of ginger supplementation showed no significant change in BW of type 2 diabetes patients (WMD = − 0.02 kg; 95% CI: −0.15, 0.10; *p* = 0.693), with no evidence of heterogeneity among studies (*p* = 0.992, I² = 0.00%).

Detailed subgroup analyses are presented in Supplementary Table S2, including heterogeneity statistics. Subgroup analyses stratified by BMI, intervention duration, and ginger dose showed no significant differences.

Meta-regression analyses revealed no association between effect size and dose (Coefficient = − 0.32; 95% CI: −1.46, 0.80; *p* = 0.571) or duration (Coefficient = 0.02; 95% CI: −0.17, 0.23; *p* = 0.782). Detailed meta-regression outputs and dose- and duration-response plots are provided in Supplementary Table S3 and Supplementary Figure S1.

Begg’s test indicated no evidence of publication bias (*p* = 0.945), whereas Egger’s test suggested possible small-study effects (*p* = 0.012) (Table S4).

Sensitivity analyses confirmed the robustness of the findings, with the leave-one-out analysis presented in Supplementary Figure S2.

#### Effects of ginger supplementation on BMI

**Fig. 4:**
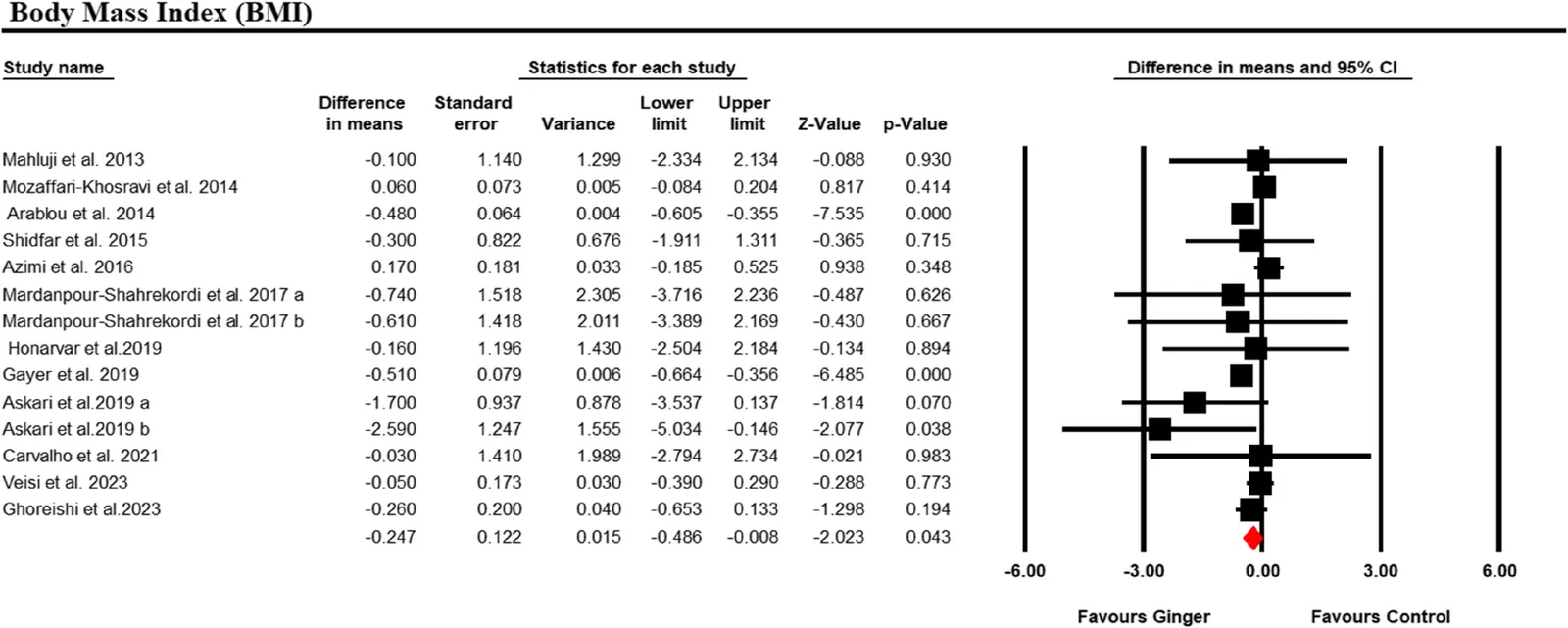
Forest plot of weighted mean difference (95% CI) for ginger effect on BMI

Figure 4 presents the forest plot for BMI, showing the WMD and 95% CIs for each study and the overall pooled estimate. The pooled effect of ginger supplementation slightly but significantly reduced BMI in type 2 diabetes patients (WMD = − 0.24 kg/m²; 95% CI: −0.48, − 0.00; *p* = 0.043), with substantial heterogeneity across studies (*p <* 0.001, I² = 75.8%).

Detailed subgroup analyses are presented in Supplementary Table S2. Subgroup analyses indicated that the significant effect was mainly observed in participants with baseline BMI > 29.9 kg/m², intervention duration > 8 weeks, and ginger doses < 2 g/day, whereas other subgroups showed non-significant effects.

Meta-regression showed a significant link between ginger dose and BMI change (Coefficient = 0.41; 95% CI: 0.29, 0.53; *p* < 0.001), but not with intervention duration (Coefficient = − 0.09; 95% CI: −0.22, 0.02; *p* = 0.124). Detailed meta-regression results and dose- and duration-response plots are provided in Supplementary Table S3 and Supplementary Figure S1.

No evidence of publication bias was observed based on Begg’s (*p* = 0.584) and Egger’s (*p* = 0.924) tests (Table S4). Leave-one-out sensitivity analysis indicated that exclusion of four individual studies (Gayar [49], Arablou [41], Askari [42], Ghoreishi [5, 44]

**Fig. 5:**
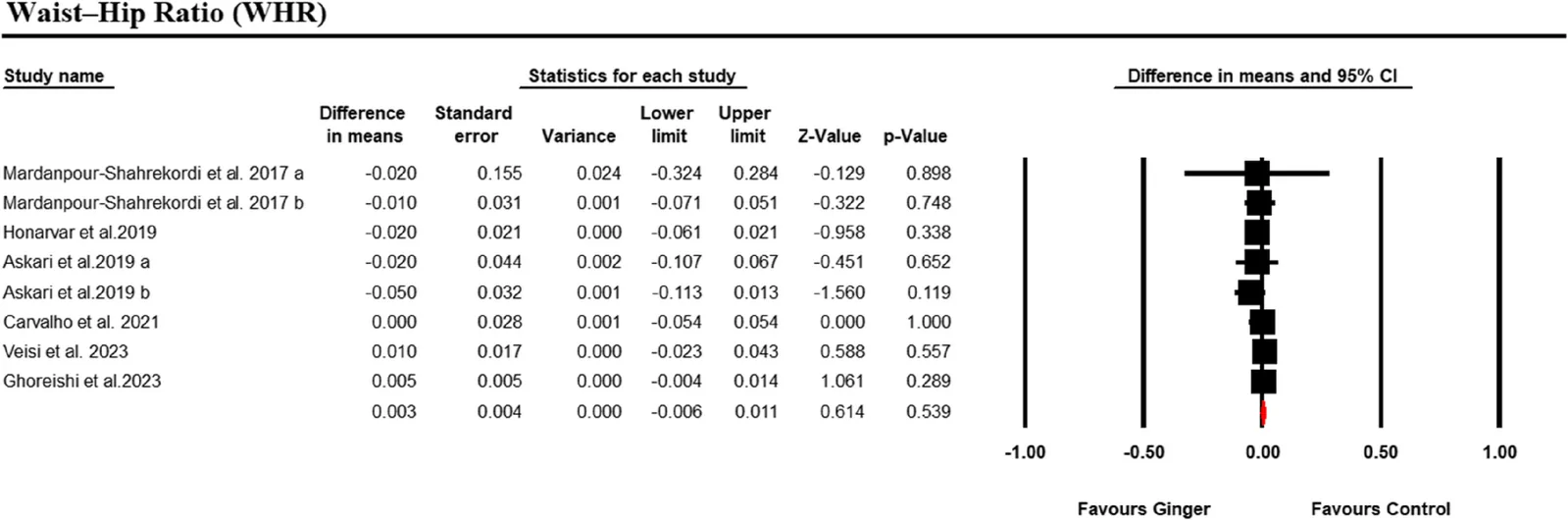
Forest plot of weighted mean difference (95% CI) for ginger effect on WHR

Detailed subgroup analyses are presented in Supplementary Table S2, showing no significant differences across baseline BMI, intervention duration, or ginger dose.

Meta-regression analyses showed no association between WHR change and ginger dose (Coefficient = 0.02; 95% CI: -0.02, 0.06; *p* = 0.349) or duration (Coefficient = 0.00; 95% CI: -0.004, 0.005; *p* = 0.875), with detailed outputs provided in Supplementary Table S3 and Supplementary Figure S1.

Begg’s and Egger’s tests showed that there was no publication bias (*p* = 0.173 and *p* = 0.081, respectively; Table S4). Sensitivity analyses confirmed the robustness of the findings (Supplementary Figure S2).

#### Effects of ginger supplementation on WC

**Fig. 6:**
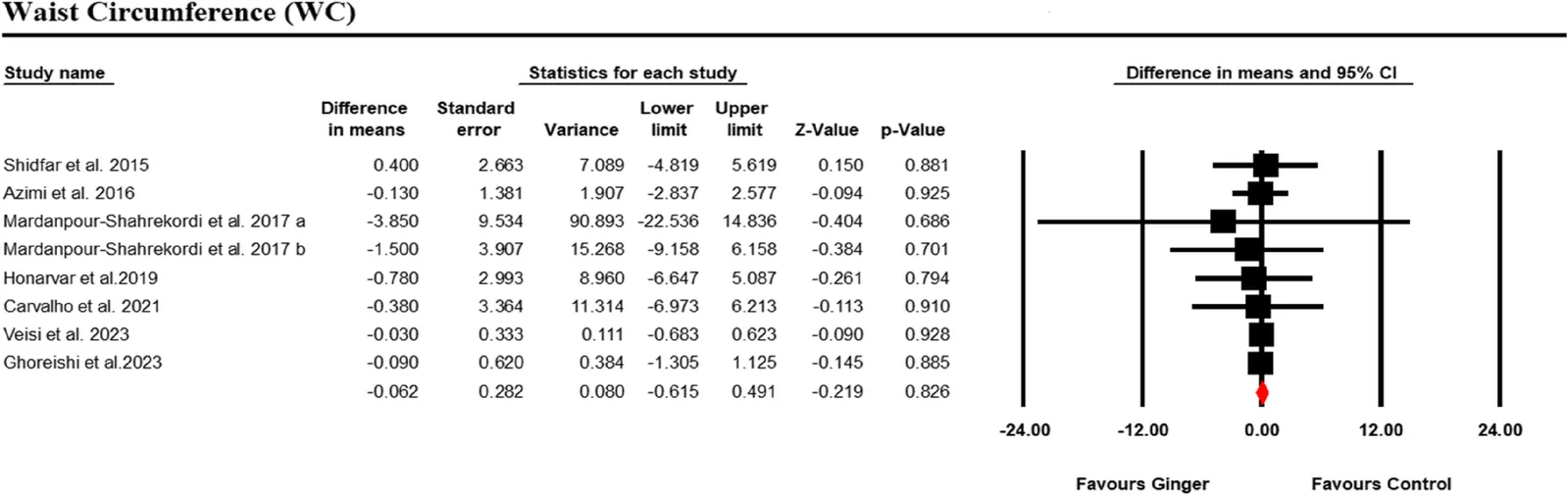
Forest plot of weighted mean difference (95% CI) for ginger effect on WC

Figure 6 presents the forest plot for WC, showing the weighted WMD and 95% CIs for individual studies and the overall pooled estimate. The pooled effect of ginger supplementation did not significantly affect WC in patients with type 2 diabetes compared with control (WMD = − 0.06 cm; 95% CI: −0.61, 0.49; *p* = 0.826), with no heterogeneity between studies (*p* = 1.00, I² = 0.00%).

Detailed subgroup analyses are presented in Supplementary Table S2. Subgroup analyses stratified by baseline BMI, intervention duration, and ginger dosage showed no significant effects or differences between subgroups.

Meta-regression showed no association of WC with dose (Coefficient = 0.24; 95% CI: -1.98, 2.46; *p* = 0.831) or intervention duration (Coefficient = -0.009; 95% CI: -0.33, 0.31; *p* = 0.953). Detailed meta-regression outputs and dose- and duration-response plots are provided in Supplementary Table S3 and Supplementary Figure S1.

Begg’s and Egger’s tests indicated the presence of publication bias (*p* = 0.018 and *p* = 0.056, respectively) (Table S4). The robustness of the results was further assessed using sensitivity analyses (Supplementary Figure S2).

#### Effects of ginger supplementation on HC

**Fig. 7:**
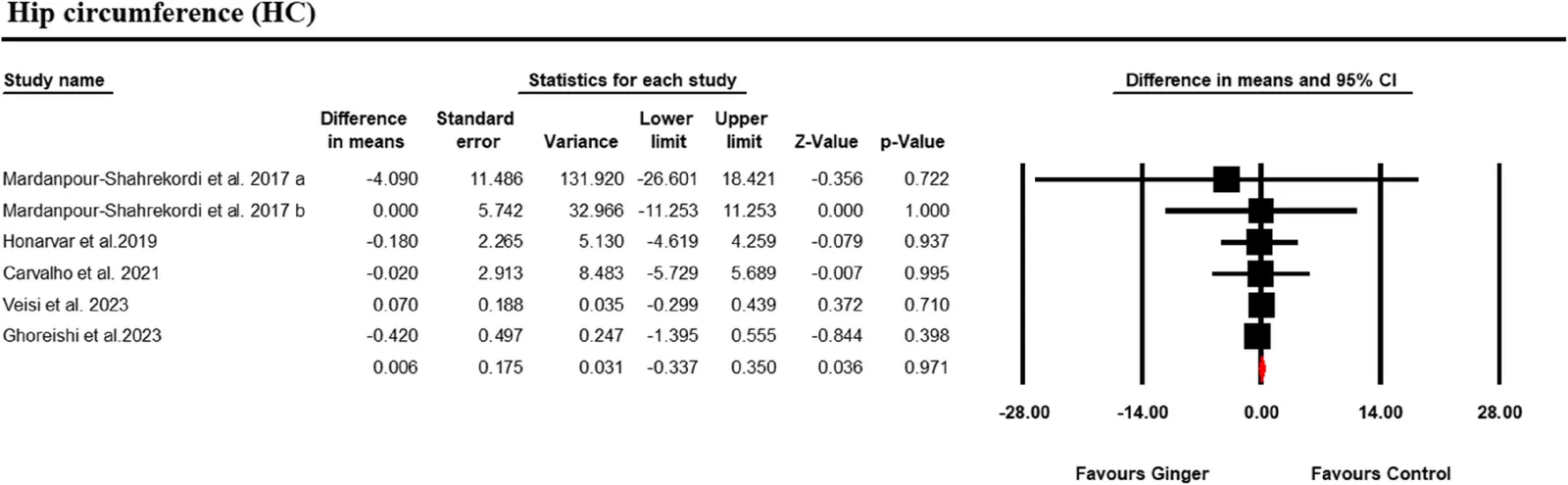
Forest plot of weighted mean difference (95% CI) for ginger effect on HC

Figure 7 presents the forest plot for HC, showing the WMD and 95% CIs for individual studies and the overall pooled estimate. The pooled effect of ginger supplementation showed no significant effect on HC in patients with type 2 diabetes (WMD = 0.006 cm; 95% CI: −0.33, 0.35; *p* = 0.971), with no heterogeneity across studies (*p* = 0.964, I² = 0.00%) (Table S2).

Subgroup analyses showed no significant differences (Table S2). Meta-regression showed no relation with dose (Coefficient = 0.29; 95% CI: -5.53, 6.13; *p* = 0.920) or duration (Coefficient = -0.11; 95% CI: -0.37, 0.13; *p* = 0.363) (Table S3; Fig. S1). No publication bias was detected (Begg’s *p* = 1.00; Egger’s *p* = 0.315) (Table S4); sensitivity analyses confirmed robustness (Supplementary Figure S2).

#### Effects of ginger supplementation on BF%

**Fig. 8:**
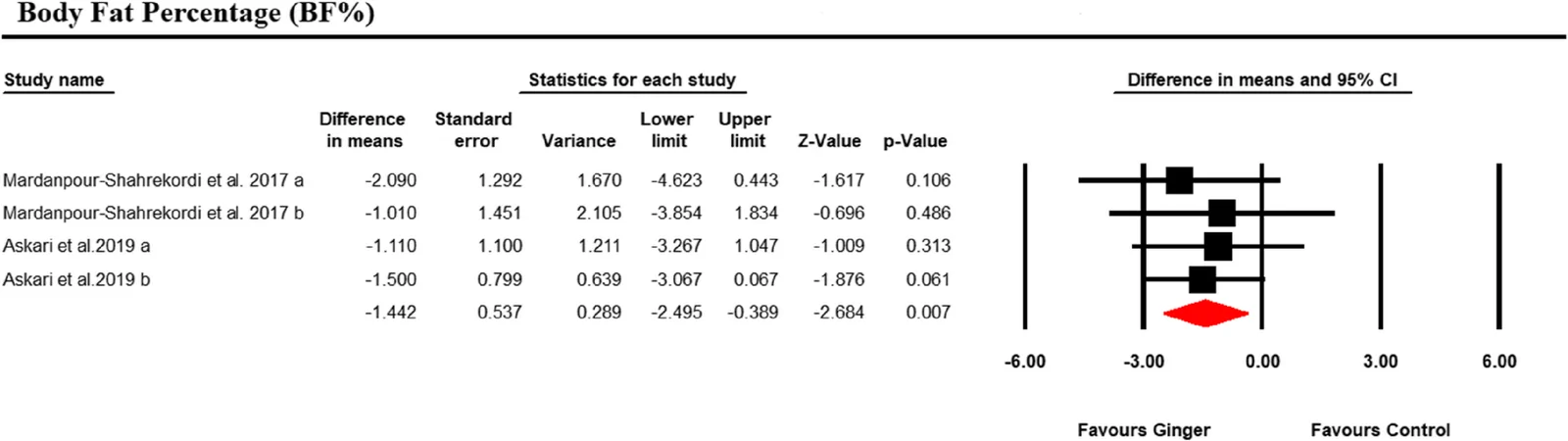
Forest plot of weighted mean difference (95% CI) for ginger effect on BF%

Figure 8 presents the forest plot for BF%, showing the WMD and 95% CIs for individual studies and the overall pooled estimate. The pooled effect of ginger supplementation shows that it significantly reduces BF% compared to control (WMD = − 1.44%; 95% CI: −2.49, − 0.38; *p* = 0.007). The reduction is consistent among studies as no heterogeneity was observed (*p* = 0.933, I² = 0.00%) (Table S2). From meta-regression, it was found that the changes in BF% were statistically not significantly dependent on the ginger dose (Coefficient = 0.41; 95% CI: -3.38, 4.20; *p* = 0.831) or intervention duration (Coefficient = 0.12; 95% CI: -1.01, 1.26; *p* = 0.831) (Table S3; Figure S1). There was no evidence of publication bias (Begg’s *p* = 1.00; Egger’s *p* = 0.918) (Table S4); the results of sensitivity analyses demonstrated the stability (Figs. S2).

### Subgroup and meta-regression analyses

Subgroup and meta-regression analyses identified sources of heterogeneity only for BMI, with baseline BMI, intervention duration, and ginger dose partially explaining the variation. No clear subgroup, dose-, or time–response effects were observed for other anthropometric outcomes. Detailed results are provided in Tables S2–S3 and Fig. S1.

### Sensitivity analysis

Leave-one-out sensitivity analyses showed that pooled estimates for BW, WC, WHR, HC, and BF% were robust. In contrast, BMI was partially sensitive, as excluding certain studies reduced the effect and made it non-significant. Detailed results are shown in Figure S2.

Additional sensitivity analyses excluding intervention arms in which ginger supplementation was combined with exercise were performed for outcomes with sufficient data. The pooled estimates for BW, WC, WHR, and HC remained materially unchanged. For BMI, the pooled effect was attenuated and was no longer statistically significant (WMD = − 0.20 kg/m²; 95% CI: −0.44, 0.04; *p* = 0.099), suggesting that the observed effect may have been partly influenced by concomitant exercise interventions. A corresponding analysis was not considered appropriate for BF% because exclusion of the exercise-combined intervention arms left only a single comparison available for analysis.

### Publication bias

Publication bias was evaluated using funnel plots, Begg’s and Egger’s tests, trim-and-fill method, and fail-safe N analysis. Following the trim-and-fill method, 0, 6, 3, 0, 3, and 4 potentially missing studies were imputed for BMI, BW, WC, BF%, HC, and WHR, respectively (Figure S3, panels A–F). Begg’s test was not significant for any outcome. Egger’s test was significant for BW (*p* = 0.012) and borderline for WC (*p* = 0.056), but not significant for the remaining outcomes (Table S4). However, the small number of trials limits the reliability of these tests.

### Certainty of evidence

GRADE assessment rated the certainty of evidence as high for BW, moderate for BMI, WHR, HC, and BF%, and low for WC (Table 2).

## Discussion

### Main findings

This meta-analysis showed that ginger supplementation led to a small decrease in BMI of type 2 diabetes patients, particularly in patients with baseline BMI > 29.9 kg/m². In addition, it significantly reduced the BF%, while changes in BW, WC, WHR, and HC were not statistically significant. The results of the subgroup analyses revealed that the decrease in BMI was associated with the initial BMI being higher, the longer the supplementation, and the daily dose being lower.

Hence, ginger may contribute to improving body composition management in T2DM patients, especially those with obesity. Nevertheless, study heterogeneity calls for additional well conducted RCTs with standardized ginger formulas, doses, and longer follow-up periods.

### Comparison with previous evidence

Systematic reviews and meta-analyses have reported mixed results on the effects of ginger supplementation on anthropometric outcomes. An extensive meta-analysis that was GRADE assessed and published in 2024 showed a significant decrease of BW, BMI, WC, and BF% as a result of ginger intake in different adult participants [18].

Consistently, a previous meta-analysis published in 2022 also reported a small yet statistically significant decrease in BMI, however, the absence of a significant effect on BW suggests a separation between overall weight change and adiposity-related indices [51].

In contrast, a 2025 systematic review indicated that ginger supplementation did not significantly impact BW or BMI, but significantly decreased WC and BF%, highlighting a potential modulation of fat distribution [52].

Randomized controlled trials in patients with type 2 diabetes have given inconsistent results; some studies have reported no changes in BMI or WC, while others have shown small improvements, which are mainly seen in short term interventions or among patients who have lower metabolic flexibility [22, 23].

Mixed study results may be due to differences in dose, duration, and participants. Higher doses (≥ 2 g/day) and longer interventions (> 8 weeks) were often linked to better anthropometric outcomes [18].

### Biological and mechanistic interpretations

Ginger supplementation may enhance body composition in patients with type 2 diabetes through multiple metabolic, inflammatory, and molecular mechanisms [25, 53, 54]. Bioactive compounds such as gingerols and shogaols promote insulin function via AMPK activation and PI3K/Akt signaling. They also augment glucose uptake and prevent adipogenic differentiation [55–57]. Additionally, inhibition of NF-κB-mediated inflammatory cascades and reduction of oxidative stress lower chronic inflammation. This leads to improved adipose tissue function and reduced ectopic fat accumulation [58–60]. Improved leptin and adiponectin signaling may contribute to fat mass reduction without altering body weight [52]. Furthermore, ginger polyphenols can influence gut microbiota and short-chain fatty acid production. This may increase metabolic flexibility and lipid oxidation [61–63]. Collectively, these mechanisms explain ginger’s specific effects on body composition rather than overall weight loss.

### Clinical and public health implications

Despite no significant effect on BW, ginger supplementation was associated with a reduction in body fat percentage, supported by moderate-certainty evidence, and a modest reduction in BMI in the primary analysis. From a clinical perspective, reductions in adiposity may be more relevant than changes in body weight alone, particularly in individuals with type 2 diabetes [64, 65]. Even modest improvements in body composition may contribute to enhanced insulin sensitivity, improved adipose tissue function, reduced systemic inflammation, and lower cardiometabolic risk [66]. Therefore, assessment of body composition, in addition to body weight, may provide a more informative evaluation of the metabolic benefits of ginger supplementation in clinical practice [67]. Nevertheless, the BMI findings should be interpreted with caution because sensitivity analyses suggested that they were partly influenced by concomitant exercise interventions.

### Sources of heterogeneity and sensitivity

Heterogeneity was observed only for BMI and appeared to be influenced by baseline BMI, supplementation dose, intervention duration, and concomitant exercise interventions. Sensitivity analyses showed that the pooled BMI effect was partially driven by individual studies and was attenuated after excluding exercise intervention arms, suggesting that the observed BMI reduction may not be solely attributable to ginger supplementation. Findings for all other outcomes remained robust.

### Limitations of the current study

There are several important limitations to consider. First, most included trials had small sample sizes and short intervention durations, limiting statistical power and the ability to infer long-term effects of ginger supplementation on anthropometric outcomes. Second, we found significant heterogeneity with respect to BMI. Although largely explained by prior subgroup analyses, the pooled BMI effect was moderately sensitive to the inclusion of individual studies, reducing confidence in this finding. Additional sensitivity analyses excluding intervention arms combining ginger supplementation with exercise further attenuated the pooled BMI effect. However, a similar analysis could not be meaningfully performed for body fat percentage because excluding the exercise-combined intervention arms left only a single comparison available for analysis.

Third, differences in ginger type, dose, and added interventions (like exercise) may have caused some variability that we couldn’t fully consider. Fourth, subgroup analyses for body fat percentage are unreliable due to few trials. Fifth, the majority of the included trials were conducted in Iran, which limits the generalizability of the findings to other populations. Differences in dietary habits, lifestyle, ethnicity, baseline nutritional status, and healthcare settings may modify the response to ginger supplementation [68, 69]. Therefore, the present findings should be interpreted with caution when applied to populations outside the geographical settings represented in the included studies.

Lastly, inconsistent reporting of adherence, dietary intake, and physical activity among trials restricts the interpretative scope for causality. Moreover, although several trials monitored dietary intake or provided dietary recommendations, participants’ short-term dietary compliance may have varied across studies. Such variation could have influenced the observed intervention effects and contributed to residual between-study variability.

### Future research directions

Future trials should move beyond conventional anthropometric endpoints and adopt mechanism-driven designs integrating adipose tissue biology, insulin signaling pathways, inflammatory and epigenetic markers, and gut microbiota profiling. Dose–response and time–response RCTs stratified by obesity phenotype, metabolic flexibility, and genetic susceptibility are warranted to identify responder subgroups. Long-term pragmatic trials examining the interaction of ginger supplementation with lifestyle interventions and standard pharmacotherapy are essential to establish clinical relevance and translational applicability.

## Conclusion

This GRADE-assessed meta-analysis of randomized controlled trials found that ginger supplementation in adults with type 2 diabetes significantly reduced BMI and BF%, while no significant changes were observed in BW, WC, HC, or WHR. The certainty of evidence was rated as high for BW, moderate for BMI, WHR, HC, and BF%, and low for WC. Although the observed reductions in BMI and BF% were modest, they may offer clinically meaningful improvements in body composition, particularly in individuals with obesity. However, further well-designed RCTs with larger sample sizes and longer follow-up periods are warranted to confirm these findings and establish optimal dosing regimens.

## Supplementary Information

Supplementary Material 1.

## Data Availability

This meta-analysis was based solely on published data; no individual-level data were collected. Extraction tables and analysis code are available from the corresponding author upon reasonable request.

## Abbreviations

T2DM: Type 2 diabetes mellitus
BMI: Body mass index
WC: Waist circumference
HC: Hip circumference
WHR: Waist-to-hip ratio
BF%: Body fat percentage
