# Supplementary material for: Effects of ginger intervention on anthropometric and body composition indices in patients with type 2 diabetes: a GRADE-assessed systematic review and meta-analysis of randomized controlled trials
Source: BMC Complement Med Ther. 2026 Aug 5;26:331. doi: 10.1186/s12906-026-05492-2 (PMC13725947; doi:10.1186/s12906-026-05492-2)
Supplement: Supplementary file 1 [file 12906_2026_5492_MOESM1_ESM.docx]

**Effects of Ginger Intervention on Anthropometric and Body Composition Indices in Patients with Type 2 Diabetes: A GRADE-Assessed Systematic Review and Meta-Analysis of Randomized Controlled Trials**

Ali Keshavarz ^1,2^, Aylee Sobhanifar ^3^**,** Parna Alizadeh ^4^, S Faezeh Hashemi Moghaddam^5^, Monirsadat Mirhoseini^6^, Fatemeh Borazjani^2,7*^

*^1^ Student Research Committee, Ahvaz Jundishapur University of Medical Sciences, Ahvaz, Iran.*

*^2^ Nutrition and Metabolic Diseases Research Center, Clinical Sciences Research Institute, Ahvaz Jundishapur University of Medical Sciences, Ahvaz, Iran.*

*^3^ Student Research Committee, Tabriz University of Medical Sciences, Tabriz, Iran.*

^4^ *Student Research and technology Committee, Faculty of Health and Nutrition, Bushehr University of Medical Sciences Bushehr, Iran.*

*^5^Department of Clinical Nutrition, School of Nutrition and Food Sciences, Shiraz University of Medical Sciences, Shiraz, Iran.*

^6^ *Department of Cellular and Molecular Nutrition, Faculty of Nutrition and Food Technology, Shahid Beheshti University of Medical Sciences, Tehran, Iran.*

^7^ *Department of Nutrition, School of Allied Medical Sciences, Ahvaz Jundishapur University of Medical Sciences, Ahvaz,Iran.*

**Table S1:** Search terms used across the various databases.

| **Database** | **Query** | **Record retrieved** |
| --- | --- | --- |
| **PubMed** | 1. "Hyperglycemia"[MeSH Terms] OR "Diabetes Mellitus"[MeSH Terms] OR "Glucose Intolerance"[MeSH Terms] OR "Diabetes Mellitus"[Title/Abstract] OR "Glucose Intolerance"[Title/Abstract] OR "diabetes"[Title/Abstract] OR "T1DM"[Title/Abstract] OR "T2DM"[Title/Abstract] OR "DM"[Title/Abstract] OR "Hyperglycemia"[Title/Abstract] OR "diabetic patients"[Title/Abstract] | 971319 |
|  | 2. "Zingiber officinale"[MeSH Terms] OR "Ginger"[Title/Abstract] OR "Zingiber"[Title/Abstract] OR "Zinziber"[Title/Abstract] OR "z officinale"[Title/Abstract] OR "Ginger supplement"[Title/Abstract] OR "Zingiber officinale"[Title/Abstract] OR "Gingembre"[Title/Abstract] | 6649 |
|  | 3. "Randomized Controlled Trial"[Publication Type] OR "Controlled Clinical Trial"[Publication Type] OR "Clinical Trial"[Publication Type] OR "Random Allocation"[MeSH Terms] OR "Single-Blind Method"[MeSH Terms] OR "Double-Blind Method"[MeSH Terms] OR "Cross-Over Studies"[MeSH Terms] OR "Clinical Trials as Topic"[MeSH Terms] OR "Comparative Study"[Publication Type] OR "Follow-Up Studies"[MeSH Terms] OR "clinical trial*"[Title/Abstract] OR "controlled trial*"[Title/Abstract] OR "intervention"[Title/Abstract] OR "Randomised"[Title/Abstract] OR "Randomized"[Title/Abstract] OR "randomly"[Title/Abstract] OR "placebo"[Title/Abstract] OR "trial"[Text Word] OR "assignment"[Text Word] OR "RCT"[Title/Abstract] OR "cross-over"[Text Word] OR "parallel"[Text Word] OR "single-blind"[Title/Abstract] OR "double-blind"[Title/Abstract] OR "triple blind"[Title/Abstract] OR "participants blinded"[Title/Abstract] OR "triple blind"[Title/Abstract] | 5674946 |
| **Final search** | (#1) AND (#2) AND (#3) | 112 |
| **Scopus** | 1. TITLE-ABS-KEY ( Hyperglycemia ) OR TITLE-ABS-KEY ( "Diabetes Mellitus" ) OR TITLE-ABS-KEY ( "Glucose Intolerance" ) OR TITLE-ABS-KEY ( diabetes ) OR TITLE-ABS-KEY ( T1DM ) OR TITLE-ABS-KEY ( T2DM ) OR TITLE-ABS-KEY ( DM ) OR TITLE-ABS-KEY ( "diabetic patients" ) | 1517967 |
|  | 2. TITLE-ABS-KEY ( Ginger ) OR TITLE-ABS-KEY ( Zingiber ) OR TITLE-ABS-KEY ( Zinziber ) OR TITLE-ABS-KEY ( "Z. officinale" ) OR TITLE-ABS-KEY ( "Ginger supplement" ) OR TITLE-ABS-KEY ( "Zingiber officinale" ) OR TITLE-ABS-KEY ( gingembre ) | 20361 |
|  | 3. TITLE-ABS-KEY("randomized controlled trial") OR TITLE-ABS-KEY("clinical trial") OR TITLE-ABS-KEY("comparative study") OR TITLE-ABS-KEY(random*) OR TITLE-ABS-KEY(placebo) OR TITLE-ABS-KEY(intervention) | 8507191 |
| **Final search** | (#1) AND (#2) AND (#3) | 358 |
| **Web of Science** | 1. TS=(Hyperglycemia) OR TS=("Diabetes Mellitus" ) OR TS=("Glucose Intolerance") OR TS=(diabetes) OR TS=(T1DM) OR TS=(T2DM) OR TS=(DM) OR TS=("diabetic patients" ) | 1095313 |
|  | 2. TS=(Ginger) OR TS=(Zingiber) OR TS=(Zinziber) OR TS=("Z. officinale") OR TS=("Ginger supplement" ) OR TS=("Zingiber officinale") OR TS=("gingembre") | 13729 |
|  | 3. TS=("randomized controlled trial") OR TS=("clinical trial") OR TS=("comparative study") OR TS=(random*) OR TS=(placebo) OR TS=(intervention) | 4993911 |
| **Final search** | #1 AND #2 AND #3 | 129 |
| **Screening** | All records(n= 599)  PubMed (n=112), ISI Web of Science (n=129), Scopus (n=358)  Duplicate: 191 | |

**Table S2.** Subgroup analyses of Ginger Supplementation on anthropometric indices and body composition

|  | Number of studies | WMD (95%CI) | *p*-value | heterogeneity | | |
| --- | --- | --- | --- | --- | --- | --- |
|  |  |  |  | *p*  heterogeneity | I^2^ | *p*  Between group |
| Subgroup analyses of Ginger Supplementation on BMI (kg/m2). | | | | | | |
| Overall effect | 14 | -0.24 (-0.48, -0.00) | 0.043 | <0.001 | 75.80% |  |
| Baseline BMI (kg/m2) |  |  |  |  |  |  |
| ≤29.9 | 8 | -0.10 (-0.42, 0.20) | 0.538 | <0.001 | 80.96% | 0.063 |
| >29.9 | 6 | -0.49 (-0.74, -0.23) | <0.001 | 0.314 | 15.52% |  |
| Trial duration (week) |  |  |  |  |  |  |
| ≤8 | 6 | -0.09 (-0.43, 0.24) | 0.576 | <0.001 | 84.62% | **0.041** |
| >8 | 8 | -0.46 (-0.58, -0.35) | <0.001 | 0.553 | 0.00% |  |
| Dose (g) |  |  |  |  |  |  |
| <2 | 7 | -0.49 (-0.59, -0.40) | <0.001 | 0.582 | 0.00% | <0.001 |
| ≥2 | 7 | 0.027 (-0.09, 0.14) | 0.651 | 0.774 | 0.00% |  |
| Subgroup analyses of Ginger Supplementation on WC (cm). | | | | | | |
| Overall effect | 8 | -0.06 (-0.61, 0.49) | 0.82 | 1.00 | 0.00% |  |
| Baseline BMI (kg/m2) |  |  |  |  |  |  |
| ≤29.9 | 5 | -0.04 (-0.66, 0.58) | 0.898 | 0.999 | 0.00% | 0.886 |
| >29.9 | 3 | -0.14 (-1.33, 1.05) | 0.819 | 0.870 | 0.00% |  |
| Trial duration (week) |  |  |  |  |  |  |
| ≤8 | 3 | -0.04 (-0.67, 0.59) | 0.905 | 0.992 | 0.00% | 0.881 |
| >8 | 5 | -0.13 (-1.28, 1.00) | 0.813 | 0.985 | 0.00% |  |
| Dose (g) |  |  |  |  |  |  |
| <2 | 3 | -1.05 (-5.88, 3.77) | 0.668 | 0.933 | 0.00% | 0.684 |
| ≥2 | 5 | -0.04 (-0.60, 0.50) | 0.864 | 0.999 | 0.00% |  |
| Subgroup analyses of Ginger Supplementation on BW (kg) | | | | | | |
| Overall effect | 12 | -0.02 (-0.15, 0.1) | 0.693 | 0.992 | 0.00% |  |
| Baseline BMI (kg/m2) |  |  |  |  |  |  |
| ≤29.9 | 7 | -0.01 (-0.13, 0.11) | 0.841 | 1.000 | 0.00% | 0.141 |
| >29.9 | 5 | -0.74 (-1.70, 0.22) | 0.131 | 0.966 | 0.00% |  |
| Trial duration (week) |  |  |  |  |  |  |
| ≤8 | 4 | -0.12 (-0.96, 0.71) | 0.774 | 0.997 | 0.00% | 0.818 |
| >8 | 8 | -0.02 (-0.14, 0.10) | 0.722 | 0.902 | 0.00% |  |
| Dose (g) |  |  |  |  |  |  |
| <2 | 6 | -0.01 (-0.14, 0.11) | 0.841 | 0.930 | 0.00% | 0.336 |
| ≥2 | 6 | -0.33 (-0.97, 0.30) | 0.308 | 0.986 | 0.00% |  |
| Subgroup analyses of Ginger Supplementation on BF%. | | | | | | |
| Overall effect | 4 | -1.44 (-2.49, -0.38) | 0.007 | 0.933 | 0.00% |  |
| Subgroup analyses of Ginger Supplementation on HC (cm). | | | | | | |
| Overall effect | 6 | 0.006 (-0.33, 0.35) | 0.971 | 0.964 | 0.00% |  |
| Baseline BMI (kg/m2) |  |  |  |  |  |  |
| ≤29.9 | 3 | 0.07 (-0.29, 0.43) | 0.717 | 0.994 | 0.00% | 0.353 |
| >29.9 | 3 | -0.42 (-1.39, 0.54) | 0.392 | 0.948 | 0.00% |  |
| Trial duration (week) |  |  |  |  |  |  |
| ≤8 | 2 | 0.07 (-0.29, 0.43) | 0.711 | 0.975 | 0.00% | 0.353 |
| >8 | 4 | -0.41 (-1.36, 0.53) | 0.394 | 0.990 | 0.00% |  |
| Dose (g) |  |  |  |  |  |  |
| <2 | 3 | -0.21 (-5.18, 4.75) | 0.933 | 0.942 | 0.00% | 0.930 |
| ≥2 | 3 | 0.007 (-0.33, 0.35) | 0.966 | 0.652 | 0.00% |  |
| Subgroup analyses of Ginger Supplementation on WHR. | | | | | | |
| Overall effect | 8 | 0.003 (-0.006, 0.011) | 0.539 | 0.686 | 0.00% |  |
| Baseline BMI (kg/m2) |  |  |  |  |  |  |
| ≤29.9 | 3 | -0.002 (-0.025 0.022) | 0.893 | 0.537 | 0.00% | 0.703 |
| >29.9 | 5 | 0.003 (-0.006. 0.012) | 0.477 | 0.497 | 0.00% |  |
| Trial duration (week) |  |  |  |  |  |  |
| ≤8 | 2 | 0.007 (-0.021, 0.036) | 0.617 | 0.757 | 0.00% | 0.739 |
| >8 | 6 | 0.002 (-0.007, 0.011) | 0.625 | 0.472 | 0.00% |  |
| Dose (g) |  |  |  |  |  |  |
| <2 | 5 | -0.018 (-0.050, 0.013) | 0.261 | 0.828 | 0.00% | 0.181 |
| ≥2 | 3 | 0.004 (-0.004, 0.013) | 0.343 | 0.475 | 0.00% |  |

(A)

(B)

(C)

(D)

(E)

**(F)**

(G)

(H)

(I)

(J)

(K)

**(L)**

**Figure S1. Dose–response and duration–response meta-regression analyses of the effects of ginger supplementation on anthropometric outcomes. Panels A–L correspond to: (A) dose–BMI, (B) duration–BMI, (C) dose–WC, (D) duration–WC, (E) dose–body weight, (F) duration–body weight, (G) dose–BF%, (H) duration–BF%, (I) dose–HC, (J) duration–HC, (K) dose–WHR, and (L) duration–WHR.**

**Table S3**. Meta-regression between changes in anthropometric outcomes and ginger dosage and intervention duration.

| **Outcome** |  | **Random effect Model** |  |
| --- | --- | --- | --- |
|  | **Coefficient** | **95% Confidence Interval** | ***p*-value** |
| **Body mass index** |  |  |  |
| Dose of supplementation (g/day) | 0.41 | (0.29, 0.53) | **< 0.001** |
| Intervention duration (weeks) | -0.09 | (-0.22,0.02) | 0.124 |
| **Waist circumference** |  |  |  |
| Dose of supplementation (g/day) | 0.24 | (-1.98, 2.46) | 0.831 |
| Intervention duration (weeks) | -0.009 | (-0.33, 0.31) | 0.95 |
| **Body weight** |  |  |  |
| Dose of supplementation (g/day) | -0.32 | (-1.46, 0.80) | 0.571 |
| Intervention duration (weeks) | 0.02 | (-0.17, 0.23) | 0.782 |
| **Body fat percentage** |  |  |  |
| Dose of supplementation (g/day) | 0.41 | (-3.38, 4.20) | 0.831 |
| Intervention duration (weeks) | 0.12 | (-1.01, 1.26) | 0.831 |
| **Hip circumference** |  |  |  |
| Dose of supplementation (g/day) | 0.29 | (-5.53, 6.13) | 0.920 |
| Intervention duration (weeks) | -0.11 | (-0.37, 0.13) | 0.363 |
| **Waist-to-hip ratio** |  |  |  |
| Dose of supplementation (g/day) | 0.02 | (-0.02, 0.06) | 0.349 |
| Intervention duration (weeks) | 0.00 | (-0.004, 0.005) | 0.875 |

*All meta-regression analyses were conducted using a random-effects model. Dose was analyzed as grams per day and duration as intervention weeks.*

**Figure S2.** Leave-one-out sensitivity analysis for the effect of Ginger Supplementation on A) BMI (kg/m2); B) WC (cm); C) body weight (kg); D) BF%; E) HC (cm); F) WHR.

**Table S4.** Publication bias

| **outcome** | **Corrected effect size** | | **Begg’s rank correlation test** | | | **Egger’s linear regression test** | | | | | **Fail-safe N test** |
| --- | --- | --- | --- | --- | --- | --- | --- | --- | --- | --- | --- |
|  | WMD | 95% CI | Kendall’s Tau | z-value | *p*-value | Intercept | 95% CI | t | df | *p*-value | N |
| BMI | -0.24 | -0.485, -0.007 | -0.10 | 0.54 | 0.584 | -0.07 | -1.72, 1.57 | 0.09 | 12 | 0.924 | 83 |
| WC | -0.04 | -0.59, 50 | -0.67 | 2.35 | 0.018 | -0.20 | -0.42, 0.00 | 2.35 | 6 | 0.056 | 0 |
| BW | -0.01 | -0.136, 0.111 | -0.01 | 0.06 | 0.945 | -0.37 | -0.65, -0.10 | 3.05 | 10 | 0.012 | 0 |
| BF% | -1.44 | -2.49, -0.38 | 0.00 | 0.00 | 1.00 | 0.11 | -4.23, 4.46 | 0.11 | 2 | 0.918 | 4 |
| HC | 0.06 | -0.25, 0.39 | 0.00 | 0.00 | 1.00 | -0.25 | -0.85, 0.35 | 1.14 | 4 | 0.31 | 0 |
| WHR | 0.004 | -0.0003, 0.013 | -0.39 | 1.36 | 0.173 | -0.68 | -1.48, 0.11 | 2.08 | 6 | 0.081 | 0 |

D

**Figure S3.** Funnel plots for the effect of Ginger Supplementation on A) BMI (kg/m2); B) WC (cm); C) body weight (kg); D) BF%; E) HC (cm); F) WHR.
